# Compostable Polylactide and Cellulose Based Packaging for Fresh-Cut Cherry Tomatoes: Performance Evaluation and Influence of Sterilization Treatment

**DOI:** 10.3390/ma13153432

**Published:** 2020-08-04

**Authors:** Marco Rapisarda, Cristina Patanè, Alessandra Pellegrino, Angelo Malvuccio, Valeria Rizzo, Giuseppe Muratore, Paola Rizzarelli

**Affiliations:** 1 CNR-Istituto per i Polimeri, Compositi e Biomateriali (IPCB)-SS di Catania, via P. Gaifami 18, 95126 Catania, Italy; marcorapis7@gmail.com; 2 CNR-Istituto per la BioEconomia (IBE), SS di Catania, via P. Gaifami 18, 95126 Catania, Italy; cristinamaria.patane@cnr.it (C.P.); alessandra.pellegrino@cnr.it (A.P.); angelo_malvuccio79@hotmail.it (A.M.); 3 Department of Agriculture, Food and Environment-Di3A, University of Catania, Via S. Sofia 100, 95123 Catania, Italy; vrizzo@unict.it (V.R.); g.muratore@unict.it (G.M.)

**Keywords:** polylactide, PET, natureflex, biodegradable film, barrier properties, packaging, mechanical properties, UV sterilization, radiofrequency, waste management

## Abstract

For food packaging, plastic materials display large appeal, mostly due to their versatility, mechanical, optical and barrier properties. However, they play an important role in environmental concerns and waste management issue. Compostable bioplastics represent alternative materials designed for a lower environmental impact. In this work, a biobased compostable packaging, constituted by polylactide (PLA) trays and NatureFlex™ film, was evaluated for fresh-cut cherry tomato. A comparative analysis was accomplished using traditional packaging materials, that is, polyethylene terephtalate (PET) trays and polypropylene (PP Coex) film. Structural stability under food contact conditions, mechanical and physical-chemical properties were investigated. Tensile mechanical properties, puncture resistance, contact angle (CA) and attenuated total reflection Fourier-transform infrared spectroscopy (ATR-FTIR), before and after UV or radiofrequency (RF) sterilization treatment, were evaluated. UV irradiation method resulted the less invasive one. Therefore, oxygen and water vapor transmission rate (OTR and WVTR), overall chemical migration test, biodegradation assessment by biochemical oxygen demand (BOD) according to ISO 14851 and disintegration test by ISO 20200 were carried out to establish the further influence of UV sterilization on the packaging. Overall, data showed that the biobased compostable packaging for a prolonged shelf-life of fresh-cut cherry tomato has better properties that were surprisingly enhanced by the UV treatment.

## 1. Introduction

Food packaging has been designed for the preservation and protection of foods from oxidative and microbial decay extending their shelf-life. Petrochemical based plastics have been increasingly used as packaging materials because of their favorable functionality characteristics such as mechanical and barrier properties to O_2_ and aroma compounds, as well as heat sealability and low cost. On the contrary, they have a very low water vapor transmission rate and remarkable amounts of these polymers are introduced in the ecosystem as industrial waste products. In fact, increased employment of plastic packages has strongly contributed to serious ecological concerns due to their persistence in the environment and limited biodegradability. At present, a wide range of biodegradable polymers has already been introduced onto the market and can now be competitive with non-biodegradable thermoplastics in different fields, including packaging.

According to standard ASTM D-5488-94d [1] and European Standard EN 13432-2000 [2], “biodegradable” is a compound that decomposes into carbon dioxide, water (in the case of aerobic degradation) or methane (in anaerobic conditions), inorganic compounds and new cell biomass. Biodegradation of plastics involves a series of processes concerning microorganisms, which use enzymes capable of depolymerizing macromolecules producing oligomeric fragments that can be incorporated inside the cells and exploited for their metabolism [3]. Biodegradation is strongly affected by the chemical structure of the polymeric material to be biodegraded and the environment in which this process occurs. Biodegradability in fact can be assessed by diverse standard tests, over a precise period and in different environments, reproducing available disposal conditions. Despite being a natural process, it is possible to manage biodegradation in an industrial environment under conditions of anaerobic composting—the result of this process is mature compost that can be used as fertilizer; anaerobic digestion (followed by stabilization through composting) will produce biogas (and therefore energy production) as well as compost. In this way, it is possible to manage solid organic waste, including manufactured substances, such as biodegradable plastic, for which the speediness of biodegradation is compatible with these processes. The requirements for industrial compostability have been established and fixed in several standards. Compostable materials must show a high level of biodegradation and disintegration on a precise and limited timescale under detailed composting conditions, without any harmful end products on the composting process or compost quality. Additionally, eco-toxicity of the compost originated from the biodegradation of the plastic items must be estimated to certify them as “compostable” [4].

Undoubtedly, biodegradable and compostable packaging (named biopackaging hereafter) offers an attractive method for environmental waste management [5]. Moreover, biobased compostable package may contribute to reduce the environmental pollution deriving from the massive use of plastics in the packaging sector since it can be wasted in the organic fraction. Additionally, legislative choice can strongly influence the widespread use of biocompostable packaging. Nevertheless, the use of compostable bioplastics in food packaging rather than conventional plastics is of course subject to the achievement of good qualitative parameters of mechanical, optical and barrier properties [6]. Among numerous kinds of biodegradable polymers, polylactide (PLA) is currently the most promising material with the brightest development prospect [7]. PLA is a thermoplastic aliphatic polyester, which is candidate to replace traditional plastics low-density polyethylene (LDPE), high-density PE (HDPE), polystyrene (PS) and polyethylene terephtalate (PET) [8,9]. PLA covers many properties that are required for food packaging industry such as good processability, transparency and high disintegration rate in compost [10]. Furthermore, PLA has good appearance, high mechanical strength, low toxicity and good barrier properties [11]. Recently, a bio-based and compostable film (NatureFlex™) has been tested as packaging for fresh-cut artichokes, potatoes and tomatoes [12,13,14,15]. Results showed that biopackaging keeps the nutritional value of tomatoes better than the traditional package, thus offering a promising alternative to reduce the impact of processing on nutrients [15]. NatureFlex™ is a range of specialty packaging films supplied by Futamura (previously, InnoviaFilms) to offer more environmentally responsible packaging material options. NatureFlex™ films are based on renewable resources and constituted by a transparent regenerated cellulose core and heat-sealable coatings on both sides, selected to provide appropriate barrier properties [16].

Many types of radiation are used for sterilization/sanitization of food and packaging like electromagnetic radiation (e.g., gamma rays and UV light) or particulate radiation (e.g., accelerated electrons). UV radiation is often used for the purposes of food preservation because inactivates many types of bacteria and viruses—DNA molecules have an absorption peak at 260 nm, therefore UV radiation causes alterations in the genes that lead to the death of microbial cells [17,18,19]. Radiofrequency (RF) heating is based on the transmission of heat to a material through the propagation of electromagnetic waves. It exploits the phenomenon of polarization affecting a dielectric material subjected to the action of an electromagnetic field. Applying an oscillating electric field, a very rapid rotational effect of the molecules is generated ensuring an instantaneous rise in temperature throughout the mass of the treated product. Some undesirable changes, involving mechanical strength, optical transparency and chemical resistance, can occur in irradiated polymeric materials [20,21].

In this work, we evaluated the performance of a biopackaging for fresh-cut cherry tomatoes. The influence of sterilization treatments was studied as well. A cellulose-based film (Natureflex™ E946, 30 μm) and PLA trays were selected and compared with a polypropylene (PP) film (20 μm) and PET trays. To evaluate the effect of UV radiation and RF to the chemical-physical characteristics of the materials chosen, tests have been performed on samples of the same type treated under UV hood or exposed to an oscillating electric field (RF). Mechanical properties, attenuated total reflection Fourier-transform infrared spectroscopy (ATR-FTIR) and wettability were monitored before and after UV and RF treatments. Some parameters are normalized respect to the film thickness (tensile mechanical properties and permeability) or are limited to the surface (contact angle) and a comparison between the two kinds of packages was accomplished. However, the penetration of irradiation can be different in diverse materials. Accordingly, we studied the performance and the resistance to the sterilization treatment of each system, comparing the selected property of the film or container before and after the irradiation. Additionally, biochemical oxygen demand (BOD), water vapor and oxygen transmission rate, overall chemical migration and disintegration measurements were carried out on the biopackaging to check the influence of UV on biodegradation. To our knowledge, this is the first study detailing the effects of sterilization methods on the performance and properties of a biopackaging system.

## 2. Materials and Methods

### 2.1. Materials

NatureFlex™ E946 film (an experimental grade sample, based on regenerated cellulose, coated on both sides by poly (vinyl alcohol) and polyvinylidene chloride) was provided by InnoviaFilm (Cumbria, UK), named “BIO E946” throughout the text. PP film (PP Coex) was supplied by Rotocalco Mediterranea (Siracusa, Italy). Polylactide Ingeo trays were furnished by Verdiamo (Rome, Italy). Tecnofoodpack S.p.A. (Pavia, Italy) supplied the PET trays. Cherry tomatoes (local landrace “Locale di Vulcano” of long-storage tomato) from the germplasm collection at CNR-IBE (Catania, Italy) were used for the experiment. Compost used both for the BOD and disintegration tests was provided by Biochemical Agro S.r.l. (Catania, Italy).

### 2.2. Procedure for Fresh-Cut Tomato Sample Preparation

Cherry Tomatoes were first washed in tap water to remove dirt; surface was disinfected for 10 min in chlorinated water and then tomatoes were cut in halves and packaged in two types of packaging systems:

(a) conventional package (CP)—truncated pyramid-shaped PET tray (thickness = 23 μm) packed into PP Coex film bags (thickness = 20 μm);

(b) biopackage (BIO)—truncated cone-shaped compostable bioplastic tray (polylactide-PLA Ingeo) (thickness = 27 μm) packed into compostable film bags (thickness = 30 μm).

Each tray was filled with ~100 g of fresh-cut tomatoes. Polymer films were hermetically sealed by a heat-sealing packaging machine (TIS400 TG, Cibra Nova s.n.c., Cernusco sul Naviglio, Milan, Italy). All samples were stored at 4 ± 0.5 °C up to 18 days in darkness.

### 2.3. Sterilization Methods

The UV irradiation was performed on films and trays by cycles of 24 h under UV hood (ASALAIR Mod. VERTICAL 700, λ = 254 nm). The RF treatment was carried out by Officine di Cartigliano (Vicenza, Italy) by exposing the material, in a tubular system, to an oscillating electric field of 27.12 MHz for 3 min at 9000 V.

### 2.4. Contact Angle Measurements

Surface wettability of original (labelled TQ), UV and RF treated compostable and traditional films was studied through static water contact angle (CA) measurements. The CA values were determined at room temperature (23 ± 2 °C) by sessile drop method on an OCA15EC (DataPhysics Instruments GmbH, Filderstadt, Germany) equipped with the SCA 20 analysis software. Small strips (5 cm × 1 cm) of the films were cut and dried in an oven for 24 h at 30 °C before the measurements, ten per sample. The films were kept flat by using a film sample holder, which allows their horizontal positioning and stretching. Water drop (2 μL) was placed with a micro syringe on the sample surface and the CA was measured within 5 s.

### 2.5. Mechanical Properties

Mechanical properties and puncture resistance were carried out on original (TQ), UV and RF treated compostable and traditional films. Mechanical properties were performed at room temperature using a tensile testing machine (Zwick/Roell Z050 model, ZwickRoell Group, Ulm, Germany) in agreement with ISO 527-1 and 527-3 standards [22,23]. The tests were accomplished on at least five film samples for each material, with a cross-head speed of 20 mm/min and a load cell of 1 kN at an initial gauge length of 100 mm. Average tensile strength (σ_M_), percentage elongation at break (ε_B_ %) and Young’s modulus (E) were calculated from the resulting stress-strain curves.

### 2.6. Puncture Resistance

The puncture resistance was carried out in agreement with UNI EN 14477:2004 [24] standard to evaluate the force required for and the deflection distance travelled by the test sample as it is penetrated by a probe with a tip of 0.8 mm in diameter, at a speed of 10 mm/min. The tests were performed on eight films per material, at room temperature using a tensile testing machine (Zwick/Roell Z050 model, ZwickRoell Group, Ulm, Germany).

### 2.7. ATR-FTIR Analysis

ATR-FTIR spectra were collected by a Spectrum Two^TM^ FT-IR spectrometer (Perkin-Elmer, PerkinElmer Inc., Waltham, MA, USA) using the Spectrum^TM^ 10 software. Spectra were measured with 8 scans and 4 cm^−1^ resolution in the range between 4000 cm^−1^ to 400 cm^−1^. The instrument is equipped with the Universal ATR accessory, a pressure arm with a force indicator, which allows the live reading of the force in the Spectrum software ensuring a good contact with the crystal before the scan.

### 2.8. Overall Migration Test

The overall migration tests were accomplished according to “Regulation (EU) N. 10/2011” [25] on trays of PET and PLA, treated under UV hood for 24 h and not. Three replicates of trays, UV treated and not, have been filled with the food simulant, closed with glass lids and incubated at 20 °C for 10 days, simultaneously to two flasks containing an equal volume of simulant used as blank. The food simulant used was an acetic acid solution at 3% (w/v) that better mimics the behavior of the tomatoes (pH less than 4.5 and hydrophilic character). The temperature and the incubation period were consistent with the worst foreseeable conditions of use for our food during its shelf-life. The overall migration of non-volatile substances from the plastic specimens was determined as the mass of non-volatile residue, after evaporation of the food simulant and expressed as mg per dm^2^ of surface area exposed to the food simulant according to the following Equation (1):

(1) $$M=\frac{\left(m_{a}-m_{b}\right)\times 1000}{S},$$

where *M* is the overall migration in food simulant in mg/dm^2^ of surface area of the sample, *m_a_* is the mass, in grams, of the residue from the sample after evaporation of the simulant with which the sample has been filled; *m_b_* is the mass, in grams, of the residue from the food simulant and *S* is the surface area, expressed in dm^2^, of the sample in contact with the food simulant.

### 2.9. OTR and WVTR

Oxygen (OTR) and water vapor transmission rate (WVTR) were measured on E946 (Innovia Films, Wigton, Cumbria, UK) and PP Coex (Rotocalco Mediterranea Srl, Siracusa, Italy), before and after UV irradiation. An OX-TRAN^®^ Model 2/21 oxygen transmission rate test system (MOCON Inc., Minneapolis, MN, USA) was used. The WVTR was assessed using a MultiPerm analyzer (ExtraSolution s.r.l., Pisa, Italy). All the samples, with a surface area of 50 cm^2^, were conditioned for 5 h and analyzed at 23 °C and 48% RH.

### 2.10. Biodegradation Degree

Degree of aerobic biodegradation was determined by respirometric method according to ISO 14851:1999 [26]. Biochemical oxygen demand (BOD) in a closed respirometer was measured with an OxiTop^®^ system. BIO E946 film and PLA tray portions were treated under UV hood for 24 h and they were tested with specimens not UV-sterilized; paper was used as positive reference. All the samples were introduced in amber bottles, together with 164 mL of test medium, under magnetic stirring and placed in an incubator, at a constant temperature (20 ± 1 °C) for about 3 months. The medium consisted of salts dissolved in water and an appropriate volume of inoculum. The salts guarantee the right amount of nutrient for microorganisms and maintaining the pH at a value of 7.4; the inoculum was obtained by filtering the supernatant of a suspension of 10 g of mature compost in 100 mL of test medium.

The degree of biodegradation was calculated according to the Equation (2):

(2) $$Biodegradation\;\left(\%\right)=\frac{BOD_{s}\times 100}{ThOD},$$

where *ThOD* is the theoretical oxygen demand, in mg/g of test material, *BOD_s_* is the specific BOD, in mg/g of test material (Equation (3)):

(3) $$BOD_{s}=\frac{BOD_{t}-BOD_{b}}{\rho_{t}}$$

and *BOD_t_* is the BOD of the flask containing the test material, in mg/L, whereas *BOD_b_* is BOD of the blank flask, in mg/L and *ρ*_t_ is the concentration of the test material in the flask, in mg/L.

### 2.11. Disintegration Test

Disintegration study was carried out on BIO E946 film and PLA tray portions, UV-treated and not, according to ISO 20200:2015 [27]. Therefore, the disintegration degree was determined under simulated composting conditions in a laboratory-scale test at 58 °C, 50% of humidity and in aerobic conditions using a synthetic compost prepared by mixing different components (sawdust 40%; rabbit-feed 30%; mature compost 10%; corn starch 10%; sugar 5%; vegetable oil 4%; urea 1%). The components of the synthetic medium were previously conditioned at 105 °C to determine the dry weight. They were subsequently mixed and added with water to obtain a mixture consisting for 55% by water and 45% by other constituents. The samples were cut into squares of size 2.5 cm × 2.5 cm (thickness < 5 mm) and, before mixing with the synthetic compost, they were dried in an oven at 40 ± 2 °C for the time needed to constant weight. The test was carried out in PP containers (dimensions of 30 cm × 20 cm × 10 cm (l, w, h)) equipped with a sealed cover, to ensure the maintenance of humidity inside; two holes in the sides for gas exchange were made. The containers were weighed before and after filling, to refill the amount of water needed to maintain the reactor humidity levels according to the normative. In each flask, 1000 g of synthetic compost, mixed with 5 g of sample, were placed on the bottom, forming a homogeneous layer. The containers were placed in an air-circulation oven at a constant temperature of 58 ± 2 °C for 90 days. The aerobic conditions were guaranteed by hand mixing the soil. At the end of the test, the flasks without the lids were placed in an oven at 58 ± 2 °C for 48 h to dry the content. Then, the compost mass and tested material were sieved using standard sieves according to ISO 3310:2016 (sieves with 10 mm, 5 mm and 2 mm span) [28]. The residues of samples that did not pass through the sieves were collected, cleaned to remove the compost and dried in an oven at 40 ± 2 °C to constant weight. The degree of disintegration was calculated according to the Equation (4):

(4) $$\mathrm{Disintegration}\;\left(\%\right)=\frac{\left(m_{i,sample}-m_{f,sample}\right)\times 100}{m_{i,sample}},$$

where $m_{i,sample}$ and $m_{f,sample}$ represent the initial sample mass and the final dry mass of the sample recovered after sieving, respectively. Photographs of recovered samples were taken for visual comparison.

### 2.12. Statistical Analysis

Contact angle and mechanical properties data were subjected to a 2-way analysis of variance (ANOVA) using CoStat version 6.003 software (CoHort Software, Monterey, CA, USA), considering “package” (“Pk”) and “sterilization treatment” (“St”) as sources of variation and their interactions. Means, when significant, were separated using the Least Significant Differences (LSD) test at a 5% level of significance. Correlations between each individual packaging vs. sterilization treatment were estimated and coefficients were evaluated for significance using CoStat version 6.003 software (CoHort Software, Monterey, CA, USA).

## 3. Results and Discussion

### 3.1. Contact Angle Measurements

**Figure 1:**
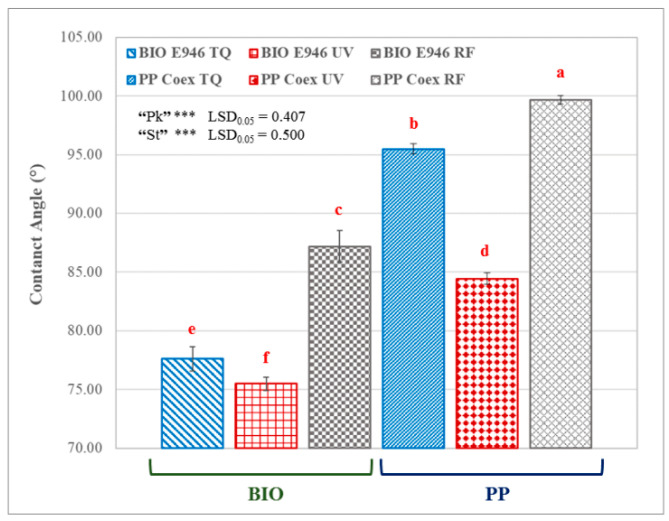
Effect of packaging film and sterilization treatment on contact angle. TQ = original sample, UV = UV treated film; and radiofrequency (RF) = RF treated sample. Different red letters above bars indicate significant differences at *p* < 0.05 (LSD test). *** Main effects significant at *p* ≤ 0.001. Error bars are also reported.

The CA is an important parameter in evaluating the hydrophilicity of materials since a low value of the static CA is correlated to a low interface tension. A surface with high oxygen-contained groups could be associated to an increase of the wettability of film due to formation of hydrogen bond [29,30] on the contrary a decrease of the wettability can be related to an increase in superficial roughness of the films [30,31]. The effect of UV and RF modification on the CA of BIO E946 and PP Coex is shown in Figure 1.

In the case of the BIO E946, similar CA values were found for both the untreated and the UV treated film samples. On the other hand, the effect of RF significantly reduced the wettability of the film, a symptom of a more marked modification of the surface (Figure 1, “St” significant, *p* < 0.05). Moreover, PP Coex films showed a marked decrease in the CA because of the exposure to UV radiation. This phenomenon is due to the formation of radical groups induced by the absorption of energy that paves the way to reactions with atmospheric oxygen for the formation of polar groups on the surface of the film [32]. Furthermore, RF treatment produced an increase in the average value of the CA in PP Coex films. The similar behavior of wettability following the RF treatment for both films, biodegradable and traditional ones (“Pk” significant, *p* < 0.05), could be due to an increase of the surface roughness [30,31]. Furthermore, in BIO E946 it could be related to a concurrent removal of the polar groups (in particular -OH of the coating) arising by heating of the sample recorded for the biodegradable sample. This could have caused an increase in the carbon/oxygen ratio with consequent greater hydrophobicity of the biodegradable film. Overall, significant interaction “St” x “Pk” was observed at ANOVA (*p* < 0.05). The treatment undergone by the materials caused similar trend of changes. The BIO film showed a more hydrophilic character than the traditional one but for both films, the UV radiation was responsible for an increase in their wettability compared to the untreated film, reasonably due to oxidation processes. Instead, the RF treatment made them more hydrophobic than TQ and UV-treated films.

### 3.2. Surface Chemical Composition Analysis

#### 3.2.1. ATR-FTIR of BIO E946

**Figure 2:**
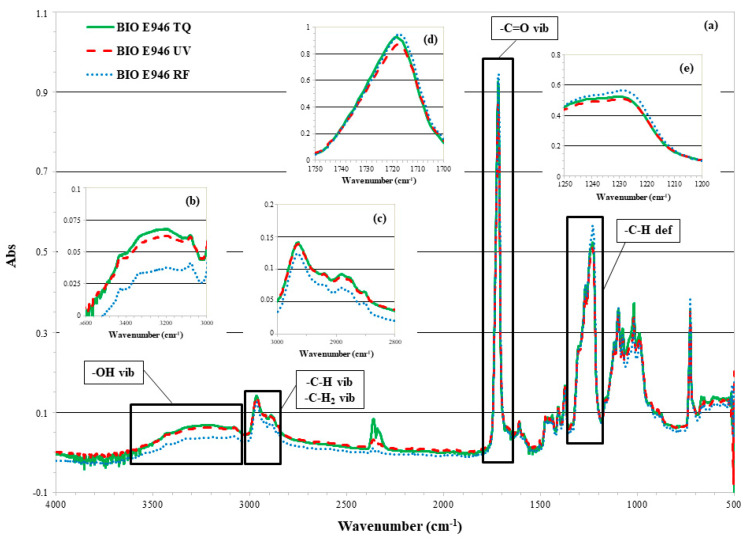
ATR-FTIR spectra of (**a**) BIO E946, before (TQ) and after UV and RF treatments; expansions in the regions (**b**) 3600–3000 cm^−1^, (**c**) 3000–2800 cm^−1^, (**d**) 1750–1700 cm^−1^ and (**e**) 1250–1200 cm^−1^.

The ATR-FTIR spectra of BIO E946 and some expansions are shown in Figure 2a–e. The large band between 3600 and 3000 cm^−1^ (Figure 2b) is due to the vibration of hydrogen bonded OH-groups, while in the range 3000–2800 cm^−1^ (Figure 2c) are recognizable the characteristic peaks of C-H and C-H_2_ asymmetrical and symmetrical stretching vibration respectively. The peak at 1720 cm^−1^ (Figure 2d) indicates the C=O valence vibration in ester groups, whereas the peaks at 1270 and 1230 cm^−1^ (Figure 2e) are attributed to C-H deformation and to C-OH out-of plane deformation respectively. The peaks at 1100, 1075, 1016 and 990 cm^−1^ are due to C=O and C=C ring vibration [33,34,35,36].

After the RF irradiation, the intensity of the peaks decreases in the wavenumber range 3600–3000 cm^−1^ (Figure 2b) and 3000–2800 cm^−1^ (Figure 2c), while increases in the wavenumber range 1750–1700 cm^−1^ (Figure 2d) and 1250–1200 cm^−1^ (Figure 2e). The increase in the wavenumber range 1750–1700 cm^−1^ for the RF-treated sample (Figure 2d) can be explained with the modifications that cellulose undergoes because of the radiations, such as oxidation of the aldehyde end-groups to carboxyl groups or oxidation of the primary alcohol groups to the aldehyde or carboxyl groups. Related to this is the decrease in absorption peaks in the region of the OH-groups, in agreement with the CA increase (Figure 1). The UV radiation causes a less significant changes to the sample. The IR spectrum is like that of the untreated one with a slight decrease in the carbonyl (Figure 2c) and OH-groups regions (Figure 2b). The minor variations for the UV treated sample may be due to the different interactions that the film presents with the two sanitization techniques used. In the case of RF there was a very marked heating of the film (around 85 °C) probably due to its hygroscopicity. The water on the surface of the film interacted with the oscillating magnetic field causing the temperature increase recorded during the treatment and, on a macroscopic level, it involved the opacification of some portions of the film. The effect of temperature could have induced structural changes highlighted by ATR-FTIR analysis.

#### 3.2.2. ATR-FTIR of PP Coex

**Figure 3:**
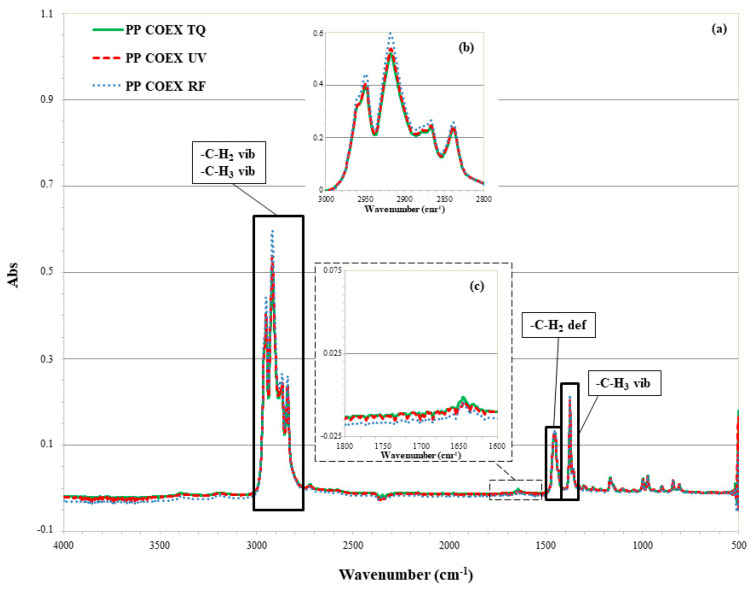
ATR-FTIR spectra of (**a**) PP Coex, before (TQ) and after UV and RF treatments; expansions in the regions (**b**) 3000-2800 cm^−1^ and (**c**) 1800-1600 cm^−1^.

The ATR-FTIR spectra of PP Coex are shown in Figure 3a. At 2950 and 2865 cm^−1^ are detectable the characteristic peaks of CH_3_ asymmetrical and symmetrical stretching vibration, respectively. The 2918 and 2840 cm^−1^ peaks can be attributed to the C-H_2_ asymmetrical and symmetrical stretching vibration, respectively. The peak at 1454 cm^−1^ points out the asymmetrical deformation of C-H_2_, while the peak at 1376 cm^−1^ is due to the symmetrical deformation of C-H_3_. The less intense peaks at 1167, 997, 841 cm^−1^ derive from C-H out of plane deformation, indicative of isotactic form [37,38,39,40,41].

The intensity of the peaks in the wavenumber range 3000–2800 cm^−1^, after the RF irradiation of the film, has undergone major changes. In particular, the RF irradiation caused greater absorption in all peaks taken in examinations in this wavenumber range (Figure 3b). This result can be explained with recombination and disproportionation phenomena with the formation of C=C bonds supported by an increase in the absorption peak at 1455 cm^−1^. No significant difference in the region between 1800 and 1600 cm^−1^ was detected (Figure 3c). This can be explained by an absence of oxidative phenomena.

### 3.3. Tensile Mechanical Properties

**Figure 4:**
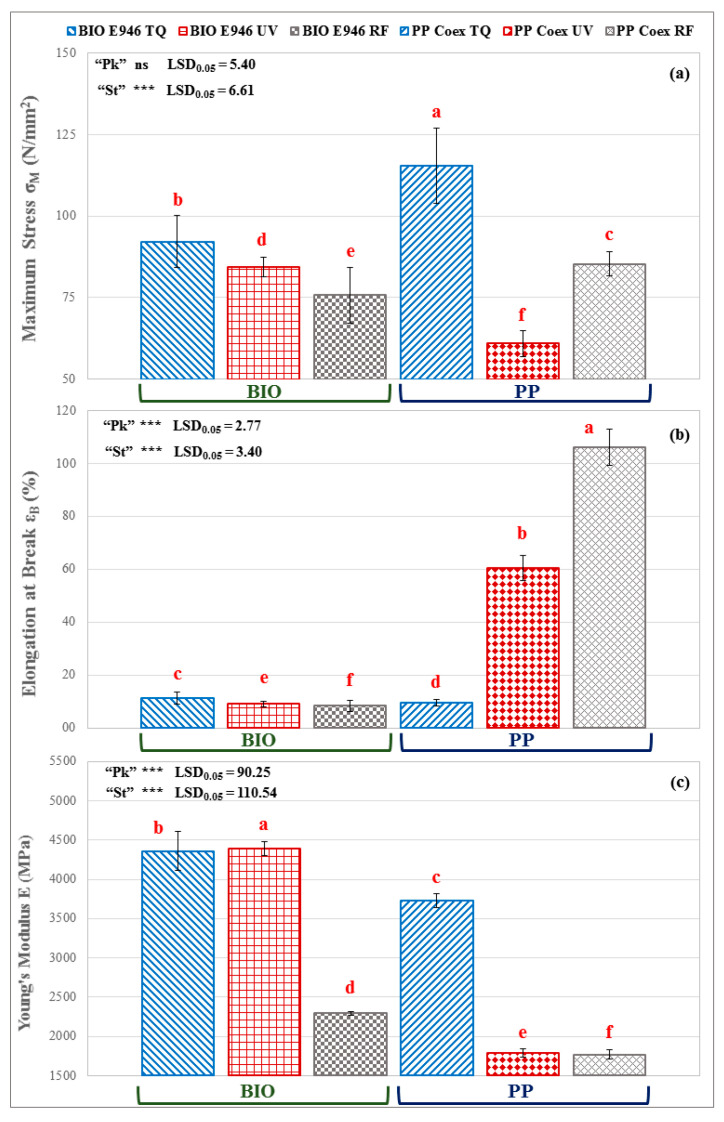
Effect of packaging film and sterilization treatment on (**a**) maximum stress, (**b**) elongation at break and (**c**) Young’s modulus of. TQ = original sample, UV = UV treated film; and RF = RF treated sample. Different red letters above bars indicate significant differences at *p* < 0.05 (LSD test). *** Main effects significant at *p* ≤ 0.001. ns = not significant. Error bars are also reported.

The values of the elongation at break (ℇ_B_), maximum load (σ_M_) and elastic modulus (E) of both films, compostable and traditional ones, original (TQ) and sterilized, are listed in Table 1. The traditional PP Coex and biodegradable BIO E946 film samples showed comparable values for the maximum stress (Table 1, “Pk” not significant, *p* > 0.05), while for the elongation at break and Young’s modulus the differences between the two types of packaging were more relevant (Table 1, “Pk” significant, *p* < 0.05). Overall, RF-induced changes were more significant than that due to UV sterilization (Figure 4). Data highlight that the RF treatment affects the maximum stress and elongation at break of the biodegradable polymers much more than the UV irradiation (Figure 4b), with an increase in the brittleness of the material (Figure 4c), due to the formation of crosslinks [42]. In PP Coex films, mechanical properties showed very evident changes due to sanitization treatments (Table 1, “St” significant, *p* < 0.05). Both the UV and RF treatments induced an increase in the elongation at break (Figure 4b) and a relevant decrease in the Young’s modulus (Figure 4c). The effect on ε_B_ of the RF treatment was more consistent than that of UV irradiation (Figure 4b). Whereas, the decrease of Young’s modulus induced by the UV and RF on PP samples was comparable (Figure 4c). In contrast to the biodegradable film, the effect of UV radiation and RF treatment caused an increase in the flexibility of the films in response to the applied stress (Figure 4b). This result could be related to the formation of the C=C bonds, in agreement with the FTIR analysis that suggests a structural modification with features like those of an elastomeric material. Significant interaction “St” x “Pk” were observed at ANOVA (*p* < 0.05). For both films, the mechanical properties are significantly influenced by the sanitization methods, causing a decrease in the maximum strength and Young’s modulus because of RF more markedly than UV radiation.

### 3.4. Puncture Resistance

The puncture resistance of both films, compostable and traditional ones, were evaluated according to UNI EN 14477:2004 [24]. In fact, knowledge of the puncture resistance and perforation behavior in polymeric films allows prevention of damage by penetration that can cause possible loss of barrier properties, package integrity and product quality. The travelled deflection distance is proportional to the elasticity of the sample while the penetration force correlates to the tensile strength, elasticity and thickness of the film. Therefore, all values are thickness and penetration surface area dependent. Anyway, as shown in Table 2, for both the biopackaging and the traditional one, not at all or negligible changes were found in the maximum load and elongation at break values of UV-treated film samples, whereas RF irradiation caused a decrease in the maximum load in the compostable film and an increase in both parameters in the traditional one. As reported by Shimamura et al. [43], UV radiation affects only a minimal part of the sample surface, leaving the underlying layers unchanged. This could explain the absence of changes in the behavior of the two films exposed to this treatment. RF radiation has most influenced the innermost layers causing reasonably crosslinking, particularly in the PP Coex film, increasing the breaking strength.

Overall, RF treatment affected contact angle, tensile mechanical properties and puncture resistance of traditional and biodegradable samples more than UV sterilization method. Therefore, further tests were focused only on UV sterilized samples.

### 3.5. Overall Migration into Aqueous Food Simulant

Two types of trays were selected, with the same volume but different section, for packaging of fresh-cut tomatoes. UV irradiation is one of the primary causes of polymer degradation [3,44] and can produce oligomers and monomers that can migrate into food. To determine the possible increase due to the UV treatment, as total amount of all non-volatile substances that could migrate into fresh cut products, the overall migration (M) into aqueous food simulant was measured (Table 3). Experimental data revealed that PLA did not release substances either before or after UV treatment. On the contrary, the migration phenomenon from PET was detectable and higher after the UV sterilization cycle (Table 3). However, M is considerably lower than the limit of 10 mg/dm^2^ established from the European regulation (EU) N. 10/2011 [25] on plastic materials and articles intended to come into contact with food. Accordingly, UV sterilization treatment does not impair the tomatoes contact suitability for both types of containers.

### 3.6. OTR and WVTR

The barrier properties of the two materials tested are very different from each other, predictably for the great dissimilarity in the chemical structure. The traditional PP film has high OTR values, two orders of magnitude higher than those of the biodegradable film—this involves a greater exchange of gases present inside the packaging that promotes the natural respiration of fresh products. On the contrary, the biodegradable film has a greater WVTR, that causes a great weight loss of the products [14]. As shown in Table 4, the UV sterilization process slightly increased the OTR of the PP Coex while decreased by about 20% in BIO E946. Instead, for both materials the WVTR was lower after sterilization.

**Figure 5:**
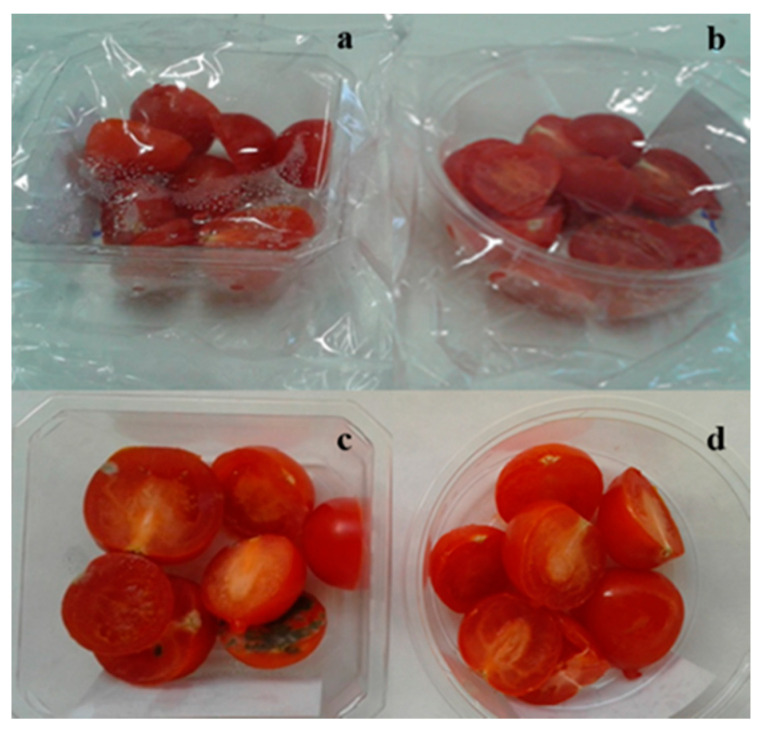
Cherry tomatoes packed in (**a**,**c**) non-biodegradable PP Coex/PET and (**b**,**d**) compostable biopackage BIO E946/PLA, after (**a**,**b**) 0 and (**c**,**d**) 18 days.

The higher WVTR of the biodegradable material ensured an anti-fog effect (Figure 5). Consequently, the product packed in biocompostable BIO E946 was unaffected by microbial growth for longer (Figure 5d), while the moisture on the inner surface of PP Coex promoted microbial growth with the appearance of molds after 18 days (Figure 5c).

### 3.7. Biodegradation Test

**Figure 6:**
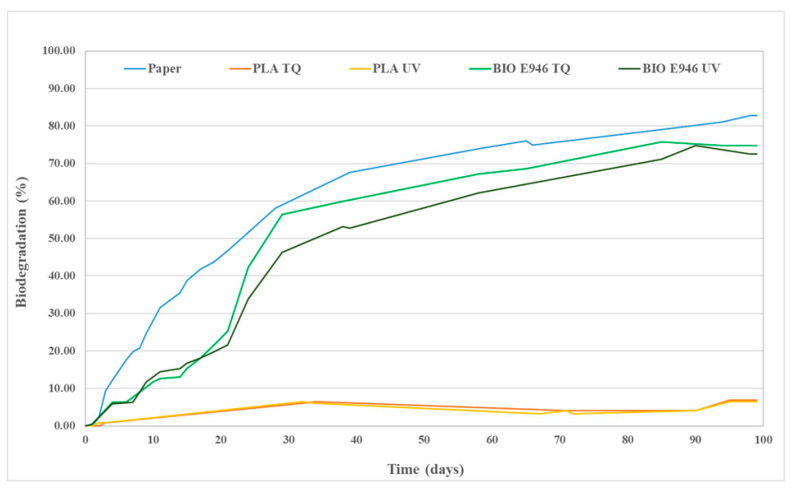
Biodegradation (%) as a function of time of PLA trays, BIO E946 film, UV treated and not (TQ) and paper (positive reference).

Biodegradation tests were carried out on BIO E946 and PLA items to check the effect of UV sterilization on biodegradation rate. A respirometric method based on BOD measurement was performed according to ISO 14851 [26]. Figure 6 displays the percentage of biodegradation (at 20 °C) plotted as a function of time of positive reference (paper), PLA trays and BIO E946, UV treated and not. The selected test temperature is the preferred one in ISO 14851. Biodegradation curves are typically characterized by—a lag phase, which is the interval from the start of the test until a clear biodegradation (i.e., 10%) can be recorded; a biodegradation phase, in which the maximum degradation takes place; and a plateau phase, in which biodegradation is almost completed. Degree of biodegradation of cellulose at the end of the test exceeded the limit value of 60%, which is required by the ISO 14851 to prove the validity of the test [26]. Predictably, BIO E946 and positive reference samples, both cellulose based materials, showed a sharp increase along the time due to the action of bacteria on the organic carbon present in the matrices. Biodegradation degree of BIO E946 was slightly affected by UV treatment. The decrease in the biodegradability of the UV treated sample may be reasonably due to crosslinking that make the film less susceptible to attack by microorganisms [45]. In agreement with the literature [46,47], biodegradation of PLA sample at 20 °C was low and the effect of UV irradiation on biodegradation degree was insignificant.

### 3.8. Disintegration Test

**Figure 7:**
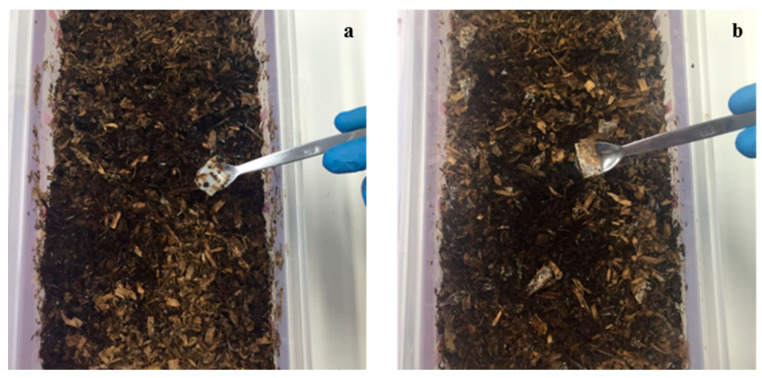
Photographic documentation of (**a**) PLA and (**b**) BIO E946 pieces after one week of disintegration test at 58 °C.

The test reproduces the humidity (about 55%) and heat (58 °C) conditions typical of a thermophilic phase of a composting plant. At this temperature, thermophilic bacteria can effectively perform their function of breaking down the organic matter contained in the samples. For each test, 5 g of BIO E946 and PLA tray portions, UV treated and not, as well as the positive references (paper) were weighed and mixed in PP containers. The fragments of BIO E946 occupied a larger volume than those deriving from the PLA trays and this has led to a more difficult dispersion in the synthetic waste mass. A week after the start of the test at 58 °C, changes in the various samples were observed. Appearance of PLA portions, UV treated and not, switched from transparent to ivory color, due to the beginning of the hydrolytic degradation, which caused a change in the refractive index of the material because of water absorption and formation of low molecular weight degradation products [48]. On the surface of BIO E946 films, traces of mold were well visible (Figure 7). Both materials started to be brittle.

At the end of the test, the compost from each container was sieved with 10 mm, 5 mm and 2 mm sieves—all pieces of test material, which do not pass through them, were collected, washed and then dried at 40 ± 2 °C in an oven. The material recovered from the sieving procedure was the non-disintegrated one, while the material which passed was considered as disintegrated. The degree of disintegration for each sample is reported in Table 5. The difference between the replicates remained below 20%, a necessary condition for the validation of the test. The results obtained show that both samples passed the disintegration test with 100% percentages in the case of PLA trays. The BIO E946 film has not reached complete disintegration, even if it has a percentage close to 100%. This slight difference could be due to the physical nature of the samples analyzed—the BIO E946 fragments have greater flexibility than that of the PLA trays causing more often the formation of lumps during the mixing phases. Lumps tend to better preserve the innermost portion from the action of microorganisms, delaying their biodegradation. For both samples examined, UV radiation did not affect the degree of disintegration. In fact, according to the standard [27], the material can be considered disintegrable when 90% of the sample weight is lost within 90 days.

## 4. Conclusions

Comparative evaluation on the performance and two different sterilization treatments confirmed that compostable polylactide and cellulose based packaging UV sterilized is the most favorable. This study shows promising results toward the use of biopackaging materials for fresh cut cherry tomatoes. Overall, the experimental data displayed that the selected biobased and compostable packaging was more suitable to sterilization treatments than conventional one. Evaluation of the structural stability under food contact conditions, mechanical and physical-chemical properties, induced by sterilization treatments, highlighted that the UV irradiation was the less invasive one. In fact, ATR-FTIR analysis, wettability and mechanical properties, carried out on both films, showed that the RF treatment caused relevant changes. BIO E946 film also displayed a direct interaction with the electromagnetic radiation resulting in a temperature increase. For both films, structural modifications did not influence their puncture resistance. Additionally, the ability to act as a barrier for oxygen remained almost unchanged for the traditional film, while it has slightly increased for the biodegradable one. WVTR decreased for both films after the UV treatment, advantageously for the BIO film sample. Furthermore, the experimental data showed that, for the biodegradable packaging system (films and trays), there were no adverse phenomena that impair their ability to biodegrade after the sterilization treatment. This work has shown that compostable films and trays can effectively replace traditional packaging without compromising product quality and shelf-life. The sanitization methods, necessary to ensure greater durability of fresh products, involve minor changes in the biopackaging. Remarkably, PLA tray and BIO film are both certified as biobased and compostable, consequently they can be wasted in the organic fraction. Therefore, the biopackaging is also helpful for the environment. In fact, the extensive employ of synthetic polymers as packaging systems is responsible of a reliable waste generation that produces several environmental pollution problems. To our knowledge, this is the first study that reports the changes induced by sterilization treatments in physical-chemical properties and biodegradation behavior of a biopackaging system selected for prolonged shelf-life of fresh-cut food.

## Figures and Tables

**Table 1:** Changes in maximum load, elongation at break and Young’s modulus in relation to package (BIO E946 = biodegradable film; PP Coex = conventional film) and sterilization treatment (TQ = original sample, UV = UV treated film; RF = RF treated sample).

Package	Sterilization Treatment	Maximum Stress σ_M_ [N mm^−2^]	Elongation at Break ε_B_ [%]	Young’s Modulus E [MPa]
BIO E946	TQ	92.1 ± 7.9	11.2 ± 2.2	4360 ± 252
	UV	84.4 ± 3.1	9.1 ± 1.0	4396 ± 89
	RF	75.7 ± 8.5	8.3 ± 2.1	2294 ± 27
PP Coex	TQ	115.5 ± 11.5	9.5 ± 1.4	3732 ± 88
	UV	60.9 ± 4.0	60.5 ± 4.7	1788 ± 54
	RF	85.3 ± 3.8	106.2 ± 6.8	1770 ± 58
Package effect (Pk)	BIO E946	84.1 a	9.5 b	3683 a
	PP Coex	87.3 a	58.7 a	2430 b
Treatment effect (St)	TQ	103.8 a	10.4 c	4046 a
	UV	80.5 b	34.8 b	3092 b
	RF	72.7 c	57.3 a	2032 c
LSD_Pk × St_ (*p* ≤ 0.05)	-	9.35	4.80	15.633

Note: Within column and main effect, average values followed by the same letter are not significantly different at *p* < 0.05 by LSD test.

**Table 2:** Maximum load and elongation at break in the puncture resistance test BIO E946 and PP Coex films, original (TQ), UV and RF treated.

**Package**	**Treatment**	**Maximum Load F [N]**	**Elongation at Break S_Max_ [mm]**
BIO E946	TQ	2.49 ± 0.12	1.18 ± 0.05
	UV	2.49 ± 0.15	1.13 ± 0.07
	RF	2.21 ± 0.14	1.19 ± 0.04
PP Coex	TQ	3.58 ± 0.07	1.68 ± 0.07
	UV	3.60 ± 0.05	1.65 ± 0.05
	RF	3.79 ± 0.09	1.83 ± 0.05

**Table 3:** Overall migration in acetic acid 3% food simulant from polylactide (PLA) and polyethylene terephtalate (PET) trays, UV treated and not (TQ).

Sample Containers			M_average_ (mg dm^−2^)
PLA (26 μm)	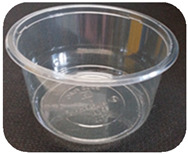	TQ	nd*
		UV	nd*
PET (23 μm)	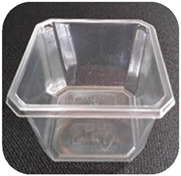	TQ	0.17
		UV	0.62

* Non detectable, below the instrument sensitivity.

**Table 4:** Oxygen and water vapor transmission rates and permeations of BIO E946 and PP Coex films, UV treated and not (TQ).

Package	Treatment	OTR (cm^3^ m^−2^ 24h^−1^)	P O_2_ (cm^3^ mil m^−2^ 24h^−1^)	WVTR (g m^−2^ 24h^−1^)	P WV (g μm m^−2^ 24h^−1^)
BIO E946	TQ	33.5	42.2	9.6	307.2
	UV	27.0	34.0	9.0	289.0
PP Coex	TQ	2292.8	1805.4	0.7	16.6
	UV	2359.6	1858.0	0.5	10.6

**Table 5:** Degree of disintegration of PLA and BIO E946, UV treated and not (TQ).

Sample	Treatment	Degree of Disintegration (%)
Trays in PLA	TQ	100.0
	UV	100.0
BIO E946	TQ	99.0
	UV	97.2
Paper	TQ	100.0
